# The Arabic Lubben Social Network Scale-6: Psychometric Validation, Measurement Invariance, and Social Support Profiles in Arabic-Speaking Older Adults

**DOI:** 10.3390/ejihpe16030040

**Published:** 2026-03-06

**Authors:** Khaled Trabelsi, Waqar Husain, Hadeel Ghazzawi, Zahra Saif, Achraf Ammar, Haitham Jahrami

**Affiliations:** 1 Research Laboratory Education, Motricité, Sport et Santé, EM2S, LR19JS01, High Institute of Sport and Physical Education of Sfax, University of Sfax, Sfax 3000, Tunisia; 2 Department of Movement Sciences and Sports Training, School of Sport Science, The University of Jordan, Amman 11942, Jordan; 3 Department of Humanities, COMSATS University Islamabad, Islamabad Campus, Park Road, Islamabad 45550, Pakistan; drsukoon@gmail.com; 4 Department of Nutrition and Food Technology, School of Agriculture, The University of Jordan, Amman 11942, Jordan; h.ghazzawi@ju.edu.jo; 5 Government Hospitals, Manama 329, Bahrain; zahra-saif@outlook.com; 6 Department of Training and Movement Science, Institute of Sport Science, Johannes Gutenberg University of Mainz, 55029 Mainz, Germany; acammar@uni-mainz.de; 7 Research Laboratory, Molecular Bases of Human Pathology, LR19ES13, Faculty of Medicine of Sfax, University of Sfax, Sfax 3000, Tunisia; 8 Department of Psychiatry, College of Medicine and Health Sciences, Arabian Gulf University, Manama 329, Bahrain

**Keywords:** social isolation, Lubben Social Network Scale, LSNS-6, Arabic validation, psychometric properties, older persons, measurement equivalence, item response theory, latent class analysis, social support

## Abstract

This study aimed to translate, culturally adapt, and validate the Arabic version of the 6-Item Lubben Social Network Scale (LSNS-6). The LSNS-6 was translated, culturally adapted, and administered, alongside the Medical Outcomes Study Social Support Survey (MOS-SSS), to 327 Arabic-speaking adults aged 60 years and older. Internal consistency was examined using Cronbach’s alpha and McDonald’s omega. Confirmatory factor analysis (CFA) tested the hypothesized two-factor structure (Family and Friends), and measurement invariance was evaluated across key sociodemographic and lifestyle variables. Convergent validity was assessed through correlations with MOS-SSS domains. Item response theory (IRT) analyses examined item discrimination and threshold parameters. Latent class analysis (LCA) explored whether the LSNS-6 could identify subgroups with distinct patterns of social connectedness and perceived support. The Arabic LSNS-6 demonstrated good internal consistency (α = 0.83; ω = 0.84) and supported the expected two-factor structure with satisfactory model fit (CFI = 0.963; TLI = 0.931; SRMR = 0.03). Convergent validity was evidenced by moderate correlations with overall perceived social support (r = 0.51). IRT analyses indicated strong discrimination for most items, and LCA identified four distinct latent classes. Overall, the Arabic LSNS-6 is a reliable and valid tool for assessing social isolation among older Arabic-speaking adults.

## 1. Introduction

Social isolation (SIL) typically refers to the objective absence or limitation of social contact with others (National Academies of Sciences, Engineering, and Medicine [NASEM], 2020). It is characterized by having few social network ties, infrequent social interactions, or possibly living alone (J. Lubben et al., 2006). In contrast to loneliness, which is a subjective feeling of being isolated, SIL is an objectively quantifiable condition based on social connectivity and interaction levels (National Academies of Sciences, Engineering, and Medicine [NASEM], 2020). It is a complex construct that includes both an objective lack of social ties and a subjective perception of loneliness or social disconnection (Cornwell & Waite, 2009). 

The increasing recognition of SIL as a significant public health concern arises from its substantial impact on mental and physical well-being (Office of the U.S. Surgeon General, 2023; National Academies of Sciences, Engineering, and Medicine [NASEM], 2020; Schoenmakers et al., 2025). Previous studies have linked SIL to increased risks of cardiovascular disease (Hodgson et al., 2020; Parker et al., 2024) and cognitive decline (Livingston et al., 2024), as well as negative psychological consequences such as increased risks of depression and anxiety (Li et al., 2024), suicidal ideation (Motillon-Toudic et al., 2022), and even premature mortality (Løvsletten & Brenn, 2024). Additionally, a study revealed that individuals who experienced SIL before hospitalization in an intensive care unit faced a greater burden of disability and elevated mortality risk within a year following their critical illness (Falvey et al., 2021). The adverse impacts are especially evident in older persons, who are more vulnerable to SIL and loneliness due to life transitions such as retirement, bereavement, or reduced physical mobility (Hawkley et al., 2022; Hawkley et al., 2019). Given these consequences, it is not surprising that SIL and loneliness impose significant financial burdens on society (WHO, 2021; Schoenmakers et al., 2025). For example, a study conducted in the United Kingdom estimated that the additional costs associated with loneliness-related health and long-term care services amounted to GBP 11,725 per person over 15 years (Fulton & Jupp, 2015). 

It should be noted that the NASEM indicated that approximately 24% of community-dwelling Americans aged 65 and older are classified as socially isolated (National Academies of Sciences, Engineering, and Medicine [NASEM], 2020). Additionally, Ran et al. (2024), in a systematic review and meta-analysis, reported a 33% prevalence of SIL among elderly individuals. This issue is likely to worsen, as the World Health Organization (WHO) highlights the rapid global growth of the population aged 60 and older, particularly in developing countries, which is expected to increase the prevalence of SIL among older adults (WHO, 2019). 

These findings indicate an immediate necessity to develop standardized screening tools and targeted interventions to identify and address SIL, particularly in older adults and those with severe health conditions. Furthermore, recognizing the critical nature of this issue, the WHO has urged governments worldwide to prioritize SIL and loneliness as major public health challenges (WHO, 2021). The WHO calls for increased political engagement and resource allocation to ensure that all individuals have access to social support networks, fostering a sense of community and solidarity (WHO, 2021). Promoting social interactions and reducing loneliness fosters healthier behaviors, enhances illness recovery, and improves overall well-being by strengthening social ties, which are linked to lower mortality rates, better health, and greater emotional resilience (Uchino, 2006; Umberson & Karas Montez, 2010; Rutledge et al., 2003; Berkman, 2003; Cassel, 1976). However, accurately assessing and understanding social networks is essential for identifying individuals at risk of SIL, implementing effective interventions to mitigate its negative effects, and evaluating the effectiveness of interventions targeting SIL (Pomeroy et al., 2023; Schoenmakers et al., 2025).

One widely used tool for assessing social networks and identifying SIL is the Lubben Social Network Scale (LSNS), which was developed based on social aging theories that recognize the essential role of family and friendships as both sources of support and facilitators of social engagement (Berkman et al., 2000; Berkman & Syme, 1979). Originally introduced in 1988 as a 10-item self-reported measure (J. E. Lubben, 1988), the LSNS has been refined into four versions: LSNS-12 (Kuru Alici & Kalanlar, 2021), LSNS-18 (Myagmarjav et al., 2019), LSNS-23 (Munn et al., 2018), and LSNS-6 (J. Lubben et al., 2006). All versions assess the frequency of social contact, network size, and the perceived support provided by these connections, making them valuable tools in gerontological research and clinical applications.

Among these versions, the six-item LSNS (LSNS-6) was specifically designed as a concise and effective assessment tool, making it particularly suitable for large-scale surveys and healthcare settings where time and resources are often limited (J. Lubben et al., 2006). Its shorter format reduces respondent burden, enhancing its feasibility for use in clinical and community-based settings, especially among older adults who may experience fatigue with longer questionnaires. 

The LSNS-6 was originally developed in English, and to ensure its applicability in different cultural contexts, it has been translated and validated in multiple languages, predominantly in Western countries, such as Chinese (Chang et al., 2018), Japanese (Kurimoto et al., 2011), Korean (Hong et al., 2011), Mongolian (Myagmarjav et al., 2019), Spanish (Vilar-Compte et al., 2018), and Portuguese (Ribeiro et al., 2012; Tavares et al., 2023). Despite its widespread adoption, a validated Arabic version of the LSNS-6 is currently unavailable, limiting its applicability among over 400 million Arabic speakers across 22 countries (ICLS, 2024). The lack of an Arabic-translated and validated LSNS-6 makes it challenging to accurately assess SIL in Arab communities, thereby restricting efforts to develop effective interventions and public health strategies. 

Furthermore, most existing validations of the LSNS-6 have relied primarily on classical test theory (CTT) approaches, focusing on scale-level indices (e.g., internal consistency, factor structure). Although CTT provides essential information about overall reliability and construct validity, it offers limited insight into how individual items function across different levels of the underlying construct. This limitation is particularly relevant for short screening instruments such as the LSNS-6, in which each item contributes substantially to measurement precision. Item response theory (IRT) provides a complementary psychometric framework by enabling item-level evaluation of discrimination and threshold parameters, thereby assessing how effectively individual items differentiate respondents across the latent continuum of social connectedness and identifying potential ceiling or floor effects (Reckase, 2006). 

In addition, SIL is not a homogeneous construct (Bu & Fancourt, 2024). Older adults may demonstrate distinct configurations of structural networks and perceived support. Variable-centered approaches evaluate latent dimensions but do not determine whether meaningful subgroups exist within the population (Berlin et al., 2014). Latent class analysis (LCA), a person-centered technique, enables identification of clinically interpretable social support profiles (Lanza & Rhoades, 2013). Incorporating LCA allows assessment of whether the LSNS-6 can move beyond aggregate total scores to stratify individuals into meaningful risk groups, thereby enhancing its relevance for targeted public health strategies.

Accordingly, the present study translated and culturally adapted the LSNS-6 into Arabic and conducted a comprehensive psychometric evaluation among older Arabic-speaking adults. Using an integrated framework combining CTT and modern approaches, we examined internal consistency, the two-factor structure (Family and Friends), measurement invariance across key subgroups, convergent validity with the Medical Outcomes Study Social Support Survey (MOS-SSS), item-level parameters using IRT, and profiles of social connectedness and perceived social support identified through LCA. 

## 2. Materials and Methods

### 2.1. The Translation Process

We employed the forward-backward translation method, as described by Beaton et al. (2000), to ensure methodological rigor and adherence to best practices in the cross-cultural adaptation of assessment tools. 

Establishing the psychometric properties of LSNS-6 in Arabic was essential to support reliable screening, cross-cultural research, and the development of targeted interventions to mitigate SIL and its associated health consequences in Arabic-speaking populations. The LSNS-6 was translated into Arabic using a rigorous approach to ensure both linguistic accuracy and cultural equivalence. Authorization for the translation was secured from the original developer of the instrument (J. Lubben et al., 2006). Initially, two bilingual experts (HJ and ZS), fluent in both Arabic and English and specializing in psycho-sociology, independently translated the original English version of the LSNS-6 into Arabic. The focus of this step was to preserve the conceptual meaning of the items rather than produce a literal translation. The two independently translated versions were then reviewed and synthesized by the research team into a single unified Arabic version, ensuring agreement on the most accurate and culturally appropriate wording for each item. The Arabic version, after being synthesized, underwent a back-translation process. Two independent bilingual experts (HG and ZS), who had no prior exposure to the original English version of the LSNS-6, translated the Arabic text back into English separately. This step facilitated comparison with the original measure to detect and address any differences in meaning or unintended semantic changes. To ensure linguistic and cultural consistency, a certified translator with expertise in health and psychological measurements conducted a comprehensive review of both the forward and back translations. This process focused on resolving inconsistencies, refining grammar, and preserving semantic integrity while ensuring alignment with Arabic language and cultural norms. Particular emphasis was placed on cultural nuances and linguistic structure to ensure that the translated scale accurately reflected the intent of the original items. Following this, an expert panel comprising psychologists, sociologists, linguists, and cultural specialists, all experienced in Arab culture and mental health, reviewed the translated items. The panel assessed the cultural relevance and sensitivity of each item, refining the language to enhance clarity, contextual appropriateness, and alignment with the constructs measured by the LSNS-6. They also ensured that the terminology was widely understood across different Arabic dialects, making the instrument accessible to a broad Arabic-speaking population.

Finally, the Arabic version of the LSNS-6 was pilot-tested with a sample of 50 Arabic-speaking older adults (≥60 years) from the general population. The primary aim of this pilot study was to assess the clarity, comprehensibility, and cultural relevance of the translated items. Participants completed the questionnaire and shared feedback regarding any challenges in understanding the items or recommendations for improvement. Based on their responses, no revisions were required, confirming that the Arabic version was well understood and appropriate for the target population.

### 2.2. The Sample, Sample Size, and Data Collection

In this cross-sectional study, participants were recruited through an online dissemination strategy using our research network across 22 Arabic-speaking countries to enhance geographic diversity. The survey link was distributed between January 2025 and March 2025 via social media platforms, including Instagram, Facebook, and Twitter/X, as well as instant messaging applications such as WhatsApp, Signal, and Viber. Members of the research team in different countries shared the invitation within professional, community, and older-adult networks. Participation was voluntary and anonymous, and eligibility criteria included being aged 60 years or older and able to read Arabic. Although recruitment targeted 22 countries, participation varied across countries, and some countries yielded no responses despite dissemination efforts. Because the survey was distributed openly online, a precise response rate could not be calculated, as the total number of individuals who viewed the invitation was unknown. The survey included the Arabic version of the Lubben Social Network Scale-6 (LSNS-6) and the Medical Outcomes Study Social Support Survey (MOS-SSS), along with demographic questions covering age, biological sex, occupation, marital status, and living arrangements. Additionally, participants provided information on dietary habits, physical activity levels, and regular medication use. Participants were also asked to self-assess their general health, mental health, psychological well-being, and physical health through a series of structured questions. Mental health was evaluated with the question: “How would you rate your mental health?”, with illustrative anchors referring to feelings of anxiety, stress, and overall mood. Physical health was assessed with the question: “How would you rate your physical health?”, with illustrative anchors referring to level of activity, endurance capacity, and physical complaints such as pain or fatigue. Responses were recorded on a 5-point Likert scale ranging from very poor to excellent. 

For the validation of the Arabic version of the questionnaire, a minimum sample size of 60 participants was considered adequate for factor analysis, based on the recommendation of 5 to 10 participants per item (Tinsley & Tinsley, 1987). Nevertheless, we targeted a larger sample to improve the precision of CFA and IRT parameter estimates. A total of 327 participants completed the questionnaire, with 60 to 80 participants from each country (Bahrain [n = 80], Egypt [n = 60], Jordan [n = 61], Saudi Arabia [n = 62], and Tunisia [n = 64]). 

### 2.3. Measurements

#### 2.3.1. The Lubben Social Network Scale-6 (LSNS-6)

The Lubben Social Network Scale-6 (LSNS-6) is a validated screening tool that evaluates social network size and support through six self-reported items. It consists of two subscales: family support (Items 1–3; for example, “How many relatives do you see or hear from at least once a month?”) and friend support (Items 4–6; for example, “How many friends do you see or hear from at least once a month?”). Responses are scored on a 6-point Likert scale (0 = None to 5 = Nine or more), with total scores ranging from 0 to 30. Lower scores indicate greater SIL, with a cutoff score of less than 12 initially proposed (J. Lubben et al., 2006). However, this threshold may vary by context, as cultural norms can influence both network structure and reporting patterns (Hong et al., 2011). A Cronbach alpha of 0.83 has been reported in the original English version of LSNS-6 (J. Lubben et al., 2006).

#### 2.3.2. The Medical Outcomes Study Social Support Survey (MOS-SSS)

To assess convergent validity, participants completed the Arabic version of the MOS-SSS, which has been previously validated in the Arabic-speaking population (Cronbach’s alpha = 0.93) (Dafaalla et al., 2016). The MOS-SSS is a 19-item self-report instrument designed to assess perceived social support across four functional domains: emotional/informational support, tangible support, affectionate support, and positive social interaction. Each item is rated on a 5-point Likert scale (1 = None of the time, 5 = All of the time), with higher scores indicating greater perceived social support. Representative examples of items include: Item 2 (Emotional/Informational Support): “Someone you can count on to listen to you when you need to talk”; Item 4 (Tangible Support): “Someone to help you if you were confined to bed”; Item 5 (Affectionate Support): “Someone who shows you love and affection”; and Item 7 (Positive Social Interaction): “Someone to do things with to help you get your mind off things.” The survey also includes a single-item measure of overall social support. The MOS-SSS is widely used in health and epidemiological research to evaluate the impact of social support on physical and mental health outcomes (Dao-Tran et al., 2023).

### 2.4. Ethical Considerations

This study was conducted in accordance with the ethical principles set forth in the Helsinki Declaration of 1964 and its subsequent revisions (1975, 1983, 1989, and 1996) (World Medical Association, 2013). Ethical approval was obtained from the Ethics Research Committee of the Psychiatric Hospital, Bahrain (PREC/2024/1104). Participation was entirely voluntary, with individuals retaining the right to withdraw at any time without any repercussions. Before starting the survey, participants provided informed consent electronically, ensuring their willingness to take part in the study. Confidentiality and anonymity were strictly maintained, as no identifiable information was collected. To prevent missing data, the survey was designed so that all questions required a response, ensuring the completeness of the dataset. No monetary or non-monetary incentives were offered for participation. Additionally, data quality measures were implemented to prevent fraudulent or duplicate responses. The survey included attention checks and validation questions to confirm participant engagement and the accuracy of responses. Furthermore, response tracking techniques were used to detect click-through behaviors, allowing for a more reliable dataset. 

### 2.5. Statistical Analyses

Descriptive statistics were used to characterize the sample and summarize the results. The mean was used to represent central tendency, while standard deviation and interquartile range were applied to assess variability. Additionally, frequencies and percentages were utilized to describe the distribution of responses for categorical variables. Skewness and kurtosis values were examined to assess the normality of responses at the item level, with values close to zero indicating approximate normality.

To assess the factor structure of the Arabic LSNS-6, confirmatory factor analysis (CFA) was conducted using the maximum likelihood estimator (Goretzko et al., 2024). Based on previous research, a two-factor model corresponding to Family and Friends was assumed (Goretzko et al., 2024). Model fit was assessed using multiple indices, including the Comparative Fit Index (CFI), Tucker–Lewis Index (TLI), Root Mean Square Error of Approximation (RMSEA) with a 90% confidence interval (CI), and Standardized Root Mean Square Residual (SRMR) (Goretzko et al., 2024). Thresholds for acceptable model fit included CFI and TLI values greater than 0.90, RMSEA values below 0.08, and SRMR values below 0.08 (Goretzko et al., 2024). Factor loadings, residual variances, and R-squared values for each item were examined to assess the strength of the relationships between items and their respective latent factors (Goretzko et al., 2024).

Measurement invariance of the Arabic LSNS-6 was evaluated using multi-group confirmatory factor analysis (MG-CFA) to assess whether the two-factor structure (Family and Friends) functioned equivalently across key subgroups, including sex (male vs. female), marital status (married vs. not married), living arrangements (living with others vs. living alone), dietary habits (unwatchful, sometimes consistent, watchful), and physical activity levels (none, weekly, daily). Invariance testing proceeded hierarchically: configural invariance (unconstrained model to verify the factor structure across groups), metric invariance (constraining factor loadings), and scalar invariance (constraining both loadings and intercepts). Models were estimated using maximum likelihood. Model fit was evaluated with CFI and TLI (>0.90 acceptable), SRMR (<0.08), and RMSEA (<0.10 with 90% CI). Non-significant chi-square differences (*p* > 0.05) and minimal changes in fit indices (ΔCFI ≤ 0.01, ΔRMSEA ≤ 0.015) between nested models indicated invariance.

The convergent and discriminant validity of the Arabic LSNS-6 were assessed using several methods (Cheung et al., 2024). Average Variance Extracted (AVE) values were calculated for each subscale, with AVE > 0.50 indicating adequate convergent validity (Cheung et al., 2024). To further assess convergent validity, Pearson’s correlation coefficients (Kirch, 2008) were computed between the LSNS-6 subscales and the dimensions of the MOS-SSS. Discriminant validity was evaluated by comparing AVE values to the Maximum Shared Variance (MSV) between factors, with AVE exceeding MSV indicating good discriminant validity (Cheung et al., 2024).

The variance explained by the factors (R-squared) was calculated for all items to determine the proportion of variance explained by the latent constructs (Cheung et al., 2024). The Heterotrait-Monotrait (HTMT) ratio was used to assess discriminant validity of each factor and inter-factor relationships, with values below 0.85 indicating distinct constructs (Cheung et al., 2024).

Information criteria, including Akaike Information Criterion (AIC) and Bayesian Information Criterion (BIC), were reported for all models to compare model fit (Cheung et al., 2024; Goretzko et al., 2024). 

The internal consistency of the Arabic LSNS-6 was assessed using Cronbach’s alpha (Tavakol & Dennick, 2011) and McDonald’s omega coefficients (Stensen & Lydersen, 2022). Reliability coefficients above 0.70 were considered acceptable, while values above 0.90 indicated excellent internal consistency (Stensen & Lydersen, 2022; Tavakol & Dennick, 2011). Guttman’s lambda-2 (Woodruff & Wu, 2012) and Greatest Lower Bound (Malkewitz et al., 2023) were computed to corroborate the robustness of internal consistency estimates.

Item response theory (IRT) analysis was conducted to examine item discrimination and difficulty (Reckase, 2006). Discrimination parameters (a values) and threshold parameters (b values) were estimated for each item to assess how well items differentiated between individuals with varying levels of social connectedness (Reckase, 2006).

To identify distinct profiles of social connectedness and perceived social support among older Arabic-speaking adults, LCA was conducted using the *glca* package. The model included the total LSNS-6 score (treated as an ordinal indicator) and the four subscale sum scores from the MOS-SSS (Emotional/Informational, Tangible, Affectionate, and Positive social interaction support), all modeled as ordinal variables. Models with two to four latent classes were compared. Model selection was based on information criteria (AIC, CAIC, BIC), entropy (higher values indicate better class separation), and bootstrap likelihood ratio tests (*p*-value for absolute fit). The final model was estimated with 4 classes using maximum likelihood estimation and a fixed seed for reproducibility. Posterior class membership probabilities were used to assign individuals to their most likely class. 

All statistical analyses were performed using R software (version 4.4.2, ‘Pile of Leaves’), released on 31 October 2024. Statistical significance was set at *p* < 0.05 for all analyses.

## 3. Results

### 3.1. Demographic Characteristics and Self-Reported Health

The results of demographic characteristics and self-reported health are presented in Table 1. The study included 327 participants, with a mean age of 63.05 years (SD = 4.42, median = 63, IQR = 8.00). The majority were female (75%) and married (62%), with 79% living with others. Regarding occupational status, 66% were unemployed or retired, while 34% were employed.

Participants reported varying levels of general health, with 43% rating their general health as good, while 28% reported average general health. Cognitive health was mostly rated as good (39%) or average (31%), but 19% reported poor cognitive health. Mental health concerns were prevalent, with 31% rating their mental health as poor or very poor. Physical health was distributed more evenly, with 35% reporting average physical health, while 33% rated it as good.

Lifestyle factors indicated that 56% of participants did not engage in regular physical activity, while 10% exercised daily and 34% exercised weekly. Dietary habits were fairly balanced, with 31% reporting being watchful, 37% sometimes consistent, and 32% unwatchful. Additionally, 31% of participants reported taking regular medications.

### 3.2. Social Networks and Social Support

Table 2 presents the descriptive statistics of the social networks and social support measures. Analysis of the LSNS-6 revealed mild-moderate levels of social connectedness among participants. The total LSNS-6 score (M = 14.04, SD = 5.46, Median = 14) demonstrated a symmetrical distribution (skewness = 0.01, kurtosis = −0.27). Additionally, participants reported stronger connections with family (M = 8.06, SD = 2.87, Median = 8) compared to friends (M = 5.98, SD = 3.31, Median = 6), suggesting that family relationships constitute a more substantial component of participants’ social networks in this age group. Examination of individual LSNS-6 items revealed that participants reported the highest scores for item 1 (M = 3.66, SD = 1.1, Median = 4), which assesses the number of relatives seen or heard from at least once a month. This item also showed the strongest negative skewness (−0.92), indicating that most participants maintained regular contact with multiple family members. In contrast, items 5 and 6, which assess the availability of friends for discussing private matters and calling on for help, received the lowest scores (M = 1.80, SD = 1.23; M = 1.83, SD = 1.16, respectively), highlighting potential limitations in friendship-based support resources.

The MOS-SSS results demonstrated generally positive perceptions of available social support across domains. Overall social support scores (M = 66.38, SD = 20.52, Median = 66) showed a slight negative skew (−0.39), indicating that most participants reported moderate to high levels of perceived support.

Among the MOS-SSS subscales, emotional/informational support received the highest raw scores (M = 28.84, SD = 9.71, Median = 31), though this partly reflects the larger number of items in this subscale. Tangible support (M = 14.15, SD = 4.73, Median = 15), affectionate support (M = 10.24, SD = 3.85, Median = 11), and positive social interaction (M = 9.89, SD = 3.57, Median = 10) all demonstrated comparable patterns when accounting for the number of items per subscale. Examination of individual MOS-SSS items revealed that participants reported the highest scores for items related to having someone who shows love and affection (item 5, M = 3.69, SD = 1.3) and having someone to do things with to help get their mind off things (item 7, M = 3.7, SD = 1.31). Conversely, items assessing the availability of companions for relaxation (item 17, M = 3.26, SD = 1.31), information to help understand situations (item 18, M = 3.27, SD = 1.23), and someone to confide in (item 19, M = 3.27, SD = 1.21) received relatively lower scores.

All MOS-SSS items and subscales displayed negative skewness, ranging from −0.12 to −0.76, indicating that participants generally reported moderate to high levels of support across domains. The kurtosis values (−0.57 to −1.22) suggest a relatively flat distribution of responses, reflecting heterogeneity in participants’ social support experiences.

### 3.3. Reliability Analysis

The reliability analysis of the LSNS-6, including separate evaluations for the Family subscale, Friends subscale, and Total scale, is presented in Table 3.

The Family subscale demonstrated acceptable internal consistency, with McDonald’s ω = 0.75 (95% CI [0.71, 0.80]) and Cronbach’s α = 0.70 (95% CI [0.60, 0.75]). Guttman’s λ2 (0.72) and the Greatest Lower Bound (0.75) further supported the reliability of this subscale. Item deletion analysis indicated that removing item 1, which assesses the number of relatives seen or heard from monthly, would significantly increase internal consistency (α = 0.83), suggesting that this item may capture a distinct aspect of family-related interactions. Conversely, removing item 2 or item 3 would considerably reduce reliability (α = 0.40 and α = 0.32, respectively), highlighting their central role in measuring the Family subscale’s construct.

The Friends subscale demonstrated higher internal consistency than the Family subscale, with McDonald’s ω = 0.86 (95% CI [0.83, 0.89]) and identical values for Cronbach’s α, Guttman’s λ2, and the Greatest Lower Bound (all 0.86). The relatively narrow 95% CIs for all reliability coefficients indicate precision in these estimates. Item deletion analysis revealed that removing item 5, which assesses friends with whom private matters can be discussed, would slightly improve reliability (α = 0.87). In contrast, removing item 4 or item 6 would reduce reliability (α = 0.75 and α = 0.79, respectively), suggesting that item 5 may capture a slightly different aspect of friendship networks while maintaining the overall cohesiveness of the subscale.

### 3.4. Confirmatory Factor Analysis (CFA)

Table 4 presents the results of the CFA for the LSNS-6, examining its hypothesized two-factor structure consisting of Family and Friends subscales. 

The CFA strongly supported the two-factor structure of the LSNS-6. For the Family factor, items 2 and 3 demonstrated robust standardized factor loadings (0.84 and 0.85, respectively, both *p* < 0.001), while item 1 showed a considerably weaker loading (0.26, *p* < 0.001). This pattern aligns with the reliability analysis findings, where item 1 functioned somewhat distinctly from other Family subscale items. The Friends factor demonstrated uniformly strong standardized factor loadings across all three items, with item 4 loading at 0.72, item 5 at 0.84, and item 6 displaying the strongest loading at 0.92 (all *p* < 0.001). These robust loadings suggest that the Friends subscale items reliably measure a coherent construct of friendship-based social networks.

Analysis of residual variances offered additional information on item performance. Within the Family subscale, item 1 had a substantially higher residual variance (0.93) compared to items 2 and 3 (0.29 and 0.28, respectively), indicating that a large portion of item 1’s variance was not explained by the Family factor. In contrast, items in the Friends subscale demonstrated progressively lower residual variances, with item 4 at 0.48, item 5 at 0.30, and item 6 at 0.16. This suggests that these items, particularly item 6, were more strongly associated with the underlying Friends construct.

The overall model fit was assessed using multiple indices. The chi-square test comparing the baseline and factor models (χ^2^ = 41.53, df = 8, *p* < 0.001) demonstrated a significant improvement in fit with the two-factor structure. The CFI of 0.963 and TLI of 0.931 exceeded the conventional threshold for good fit (>0.90), along with other incremental fit indices, including the Non-Normed Fit Index (NNFI = 0.931), Normed Fit Index (NFI = 0.955), Relative Fit Index (RFI = 0.916), and Incremental Fit Index (IFI = 0.964).

The RMSEA of 0.113 (90% CI [0.081, 0.148]) exceeded the conventional threshold for good fit (<0.08), suggesting some residual misfit. However, the SRMR of 0.03 was well below the threshold (<0.08), indicating a good fit. Additional absolute fit indices (GFI = 0.992, MFI = 0.95) further supported adequate model fit.

The AVE values for both factors exceeded the recommended threshold of 0.50 (Family: 0.544; Friends: 0.673), supporting convergent validity. The HTMT ratio (0.712) between the Family and Friends factors was below 0.85, indicating adequate discriminant validity between the two subscales.

Measurement invariance was generally supported across key sociodemographic and lifestyle subgroups of the Arabic LSNS-6. Before invariance testing, model fit was evaluated separately within each group (see Table S1).

For sex, configural (CFI = 0.96, TLI = 0.93, SRMR = 0.04, RMSEA = 0.12), metric (CFI = 0.96, TLI = 0.95, SRMR = 0.04, RMSEA = 0.10), and scalar (CFI = 0.97, TLI = 0.96, SRMR = 0.04, RMSEA = 0.09) models demonstrated good fit with equivalent loadings and intercepts. Similar patterns held for marital status (configural: CFI = 0.96, TLI = 0.93, SRMR = 0.04, RMSEA = 0.12; metric: CFI = 0.96, TLI = 0.94, SRMR = 0.04, RMSEA = 0.10; scalar: CFI = 0.97, TLI = 0.96, SRMR = 0.04, RMSEA = 0.09) and living arrangements (configural: CFI = 0.96, TLI = 0.92, SRMR = 0.03, RMSEA = 0.12; metric: CFI = 0.96, TLI = 0.94, SRMR = 0.04, RMSEA = 0.11; scalar: CFI = 0.96, TLI = 0.94, SRMR = 0.04, RMSEA = 0.10). Across dietary habits, configural invariance was partially supported with borderline fit (CFI = 0.94, TLI = 0.89, SRMR = 0.05, RMSEA = 0.15), but metric (CFI = 0.94, TLI = 0.91, SRMR = 0.06, RMSEA = 0.13) and scalar (CFI = 0.94, TLI = 0.93, SRMR = 0.06, RMSEA = 0.12) models confirmed equivalence. For physical activity, all levels showed strong invariance (metric: CFI = 0.96, TLI = 0.95, SRMR = 0.05, RMSEA = 0.10; scalar: CFI = 0.95, TLI = 0.94, SRMR = 0.06, RMSEA = 0.11). Standardized loadings remained consistent across groups, with minor variations in residual variances.

### 3.5. Convergent Validity

Table 5 presents the correlational analysis assessing the convergent validity of the LSNS-6 by examining its relationship with the MOS-SSS. This analysis explores the association between structural aspects of social networks (network size) and functional aspects of social support (perceived support).

The results demonstrated moderate positive correlations between the LSNS-6 total score and all MOS-SSS subscales (r ranging from 0.43 to 0.51, all *p* < 0.001). The strongest associations were observed with overall MOS-SSS (r = 0.51, *p* < 0.001) and Emotional/Informational Support (r = 0.48, *p* < 0.001), reinforcing the LSNS-6’s validity as a measure of social connectedness.

The LSNS-6 Family subscale consistently showed stronger correlations with MOS-SSS subscales (r ranging from 0.45 to 0.52, all *p* < 0.001) compared to the LSNS-6 Friends subscale (r ranging from 0.29 to 0.41, all *p* < 0.001). This difference was particularly notable for Tangible Support, where the correlation with the Family subscale (r = 0.51, *p* < 0.001) was substantially stronger than with the Friends subscale (r = 0.29, *p* < 0.001). Similarly, Affectionate Support had a stronger association with the Family subscale (r = 0.49, *p* < 0.001) than with the Friends subscale (r = 0.29, *p* < 0.001).

In contrast, Positive Social Interaction had relatively similar correlations with both the Family subscale (r = 0.45, *p* < 0.001) and the Friends subscale (r = 0.41, *p* < 0.001), suggesting that both family and friend networks contribute similarly to this specific dimension of social support.

### 3.6. Multi-Item Response Theory (IRT) Analysis

Table 6 presents the results of the IRT analysis for the LSNS-6, detailing item discrimination and difficulty parameters within the two-factor structure.

The discrimination parameters (a1 and a2) indicate how effectively each item differentiates between individuals at different levels of the latent trait. For Factor 1 (Family), items 2 and 3 had high discrimination values (a1 = 3.45 and a1 = 3.64, respectively), suggesting these items are highly informative for assessing family-related social networks. In contrast, item 1 demonstrated notably lower discrimination (a1 = 0.38), aligning with previous findings indicating that this item functions differently from the other Family subscale items. For Factor 2 (Friends), the discrimination parameters increased progressively across items 4, 5, and 6 (a2 = 2.06, a2 = 3.33, and a2 = 12.61, respectively). The high discrimination value for item 6 suggests that this item is particularly effective at distinguishing between individuals with different levels of friendship networks, particularly near the threshold levels specified by its difficulty parameters.

The difficulty parameters (b1 through b5) represent the thresholds at which respondents have a 50% probability of endorsing each response option or higher. For most items, these parameters followed a relatively orderly progression from negative to positive values, indicating that the scale effectively measures a broad range of the underlying construct. Item 1 had the most extreme difficulty parameters, with b1 = −6.88 and b4 = 3.77, suggesting that it can differentiate across a wide range of the family network construct, albeit with limited precision due to its low discrimination value. The inversion of parameters b4 and b5 for this item (b4 = 3.77, b5 = 2.30) suggests potential response pattern irregularities at the upper end of the scale. For the remaining items, the difficulty parameters were more evenly distributed, generally ranging from approximately −1.6 to 3.0. This range indicates that the LSNS-6 provides optimal measurement precision around the middle to slightly above-average levels of the social network construct, with reduced precision at extremely low or high levels.

The covariance structure between items and factors (cov.F1 and cov.F2) followed a block pattern consistent with the two-factor model. Each item had a covariance of 1.0 with its primary factor and 0.67 with the secondary factor, suggesting a moderate correlation between the Family and Friends factors while maintaining their distinct constructs.

### 3.7. Latent Class Analysis (LCA)

**Figure 1:**
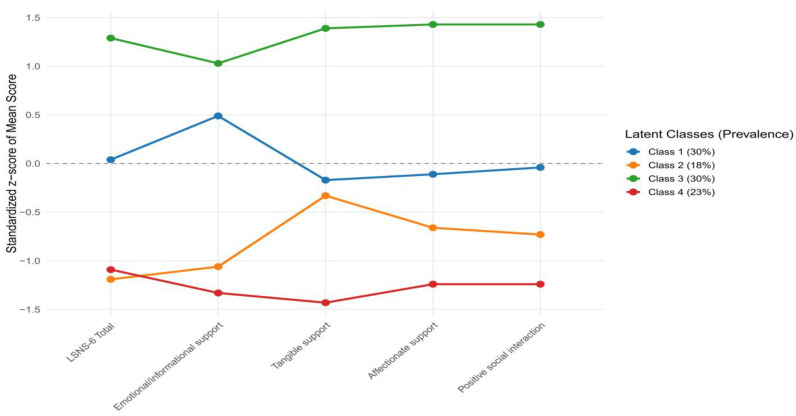
Standardized z-score profiles and class prevalences for the four latent classes across the LSNS-6 total score and MOS-SSS subscale scores (N = 327).

The LCA supported a four-class solution as the best-fitting model (AIC = 7793, BIC = 9479, entropy = 1.00, bootstrap *p* = 0.02). The estimated class prevalences were approximately 30% (Class 1), 18% (Class 2), 30% (Class 3), and 23% (Class 4). The detailed item-response probabilities, class characteristics, class prevalences, and standardized profile patterns of the four-class solution are presented in Table 7 and Figure 1.

Class 1 showed moderate-to-high LSNS-6 probabilities and consistently strong perceived support across MOS-SSS domains. The mean LSNS-6 total score was 14.11 (SD = 4.72), and overall perceived support was relatively high (MOS-SSS mean = 64.27, SD = 9.80).

Class 2 represented the lowest-support subgroup. This class had the weakest emotional/informational and affectionate support and generally lower LSNS-6 total scores. Overall functional support was reduced (MOS-SSS mean = 55.90, SD = 25.29), with substantial variability, particularly in emotional/informational support (mean = 23.52, SD = 11.66). The mean LSNS-6 total score was 12.83 (SD = 6.82).

Class 3 demonstrated the highest and most consistent support levels. Members showed the highest LSNS-6 total score (mean = 15.89, SD = 5.45) and the strongest MOS-SSS scores (overall mean = 87.79, SD = 10.63), particularly for affectionate (mean = 13.98, SD = 1.91) and emotional/informational support (mean = 37.96, SD = 4.98).

Class 4 showed a pragmatic pattern with moderate LSNS-6 total score (mean = 12.51, SD = 4.50) and comparatively lower functional support. Positive social interaction (mean = 6.89, SD = 1.76), affectionate support (mean = 7.29, SD = 2.21), and overall support (MOS-SSS mean = 49.53, SD = 10.46) were reduced despite adequate network size.

## 4. Discussion

This study presents the first validation of the Arabic version of the LSNS-6 and yields three principal findings with implications for assessing SIL in Arabic-speaking populations. First, the Friends subscale demonstrated consistently strong psychometric performance across classical test theory and IRT analyses. Factor loadings ranged from 0.72 to 0.92, internal consistency was robust (ω = 0.86), and discrimination parameters were high, particularly for Item 6 (a = 12.61). These findings indicate that friendship-based social networks are reliably and sensitively captured in this cultural context. Second, the Family subscale revealed culturally specific response patterns. Items assessing emotional closeness and family network size (Items 2 and 3) performed well psychometrically, whereas the contact frequency item (Item 1) showed weaker performance. This pattern likely reflects collectivistic cultural norms in which strong family bonds are maintained independently of frequent communication. This finding challenges frequency-based assumptions about family relationships common in Western instruments and underscores the necessity of culturally informed measurement development. Third, the identification of four distinct social support profiles, including a vulnerable 18% low-support subgroup, demonstrates that the Arabic LSNS-6 can stratify individuals for targeted intervention beyond binary isolation classifications. These findings collectively advance understanding of how social network assessment instruments must be adapted for collectivistic contexts and how brief screening tools can inform precision public health approaches.

Descriptive analysis of the LSNS-6 revealed mild-moderate levels of social connectedness among the sample, with a mean total score of 14.04 (SD = 5.46, median = 14). This value falls above the established cutoff of <12 proposed by J. Lubben et al. (2006) for identifying greater risk of SIL, indicating that the majority of participants (approximately 65%, based on the normal distribution approximation with Z = −0.374) scored at or above this threshold. The near-symmetrical distribution of scores (skewness = 0.01; kurtosis = −0.27) and substantial variability further support the interpretation of moderate but heterogeneous social networks. Stronger family ties (M = 8.06) compared with friendships (M = 5.98) are consistent with collectivistic social structures in Arabic-speaking populations. 

CFA supported the two-factor structure of the Arabic LSNS-6, distinguishing between Family and Friends subscales. This structure aligns with previous validations in other populations (Buckley et al., 2022; Jang et al., 2022; J. Lubben et al., 2006), reaffirming that family-based and friendship-based networks represent distinct but complementary aspects of social connectedness. Model fit indices, including CFI (0.963), TLI (0.931), and SRMR (0.03), indicated good overall model fit, although RMSEA (0.113) exceeded the ideal threshold. This pattern is not uncommon in CFA of brief scales with limited degrees of freedom. Several factors contribute to this phenomenon: (1) RMSEA is particularly sensitive to model complexity and degrees of freedom—short scales with few items naturally have minimal degrees of freedom (in this case, df = 8), which can lead to elevated RMSEA values even when the model is otherwise well-specified; (2) incremental fit indices (CFI, TLI) compare the specified model to a baseline model and may be less sensitive to absolute fit departures, particularly in smaller models; (3) brief screening instruments such as the LSNS-6 are necessarily parsimonious and may not capture the complexity of constructs, leading to some residual misfit; and (4) RMSEA is also known to penalize simpler models relative to more saturated models, which can inflate RMSEA values for instruments designed to be brief and efficient (Kenny et al., 2015). Given these considerations, the moderate-to-good performance on incremental fit indices and absolute fit indices (SRMR, TLI, CFI), combined with the strong factor loadings and theoretical coherence of the two-factor structure, suggests that the two-factor model provides an adequate fit to the data.

Within the Family subscale, items 2 and 3 demonstrated strong loadings (>0.80), whereas item 1, which assesses the frequency of contact with relatives, showed a considerably weaker loading (0.26). In contrast, all Friends items loaded strongly, particularly item 6 (0.92), reflecting the internal consistency of this subscale. Validity was further supported by acceptable AVE values (Family = 0.544, Friends = 0.673) and an HTMT of 0.712; this confirms adequate discriminant validity between the two subscales. 

The Arabic LSNS-6 demonstrated strong internal consistency, with Cronbach’s alpha (α = 0.83) and McDonald’s omega (ω = 0.84), both exceeding the conventional threshold of 0.70 (Revelle & Condon, 2019). These values are comparable to those reported for the original LSNS-6 (J. Lubben et al., 2006) and previous validation studies (Chang et al., 2018; Jang et al., 2022; Kurimoto et al., 2011), supporting the reliability and robustness of the scale in assessing social network size and perceived support within this sample. When examining the subscales separately, the Friends subscale demonstrated higher reliability (ω = 0.86, α = 0.86) than the Family subscale (ω = 0.75, α = 0.70). This suggests that friendship-related items were more internally consistent, whereas the Family subscale had slightly greater variability. The lower internal consistency in the Family subscale may reflect greater diversity in the structure and strength of family networks, which could be influenced by cultural and contextual factors unique to Arabic-speaking populations. Nevertheless, both subscales met acceptable reliability standards, supporting the overall reliability of the Arabic LSNS-6 in assessing social networks.

Item deletion analysis revealed that removing item 1 (which assesses the frequency of contact with relatives) would improve the reliability of the Family subscale. This finding suggests that contact frequency alone may not be a strong indicator of meaningful social support in collectivistic cultures, where strong emotional bonds often persist despite infrequent communication. Together, these results suggest that the Arabic LSNS-6 is a psychometrically robust tool, though refinements to certain items, particularly within the Family subscale, could further enhance its reliability and applicability.

A notable finding from the CFA and IRT analyses concerns Item 1, which assesses the frequency of contact with relatives (“How many relatives do you see or hear from at least once a month?”). This item demonstrated a considerably weak standardized factor loading (0.26, *p* < 0.001) within the Family subscale, along with notably lower discrimination power in the IRT analysis (a = 0.38). These psychometric properties suggest that Item 1 functions distinctly and less effectively compared to Items 2 and 3 (which assess the size of the family network and closeness of family relationships, respectively).

Item deletion analysis indicated that removing Item 1 would actually improve the internal consistency of the Family subscale from α = 0.70 to α = 0.83, suggesting that this item may not cohere well with the other family-related items. The weak loading and low discrimination suggest that contact frequency alone may not be a strong indicator of meaningful family social support in Arabic-speaking cultures, where strong emotional bonds often persist despite infrequent communication. This pattern reflects the collectivistic cultural context of Arabic-speaking populations, where family relationships are characterized by deep emotional ties and interdependence that transcend frequency of contact. In such cultures, family members may maintain strong psychological and emotional connections even when face-to-face or direct communication is infrequent due to geographic distance, life circumstances, or other practical barriers.

Examination of Item 1’s performance across previous LSNS-6 validations reveals considerable variability, with factor loadings ranging from 0.26 (present study) to 0.85. Specifically, the original English validation by J. Lubben et al. (2006) reported a factor loading of 0.77 for Item 1, indicating strong performance in the original language context. Similarly, the Chinese validation by Chang et al. (2018) demonstrated robust performance with a factor loading of 0.85, and the Mongolian validation by Myagmarjav et al. (2019) reported a loading of 0.75. However, the Korean validation by Hong et al. (2011) reported a notably lower factor loading of 0.46, intermediate between the strong performance in English, Chinese, and Mongolian contexts and the substantially weaker performance (0.26) observed in the present Arabic validation. Except for the Korean version, previous validations have consistently demonstrated moderate to strong factor loadings for Item 1 (ranging from 0.75 to 0.85), suggesting that the item functions adequately in most cultural and linguistic contexts. The Arabic version’s loading of 0.26 represents a substantial departure from this pattern and is the lowest reported to date. The Korean validation’s intermediate loading (0.46) suggests that East Asian contexts may present some challenges for this item, but the Arabic version’s performance is markedly poorer. This differential performance across languages and cultures suggests that translation-specific issues or cultural factors particular to Arabic adaptation may be responsible, rather than inherent limitations of the item itself.

Despite these psychometric limitations, we elected to retain Item 1 in the Arabic LSNS-6 for several important reasons. First, maintaining the original structure of the LSNS-6 facilitates international comparisons and allows researchers to track consistency with validations in other languages and cultural contexts. Second, removing an item would alter the instrument’s established scoring structure and potentially compromise its utility as a standardized screening tool. Third, the item still contributes meaningfully to the overall scale, and the weak loading may reflect a true cultural difference rather than a measurement error—specifically, that contact frequency may be less relevant than emotional closeness in assessing family social networks in this population.

The establishment of full measurement invariance across sex, marital status, living arrangements, dietary habits, and physical activity levels highlights the robustness of the Arabic LSNS-6 as a culturally adapted tool for assessing SIL among diverse subgroups of older Arabic-speaking adults. This equivalence ensures that observed differences in latent means accurately reflect true variations in social connectedness rather than measurement artifacts, thereby facilitating valid cross-group comparisons in future research and interventions. Notably, the slightly borderline fit for dietary habits may indicate cultural nuances in the intersection of health behaviors and social networks, necessitating cautious interpretation in nutrition-focused studies. These findings extend the scale’s utility beyond its initial validation, addressing a key limitation of previous LSNS-6 adaptations by confirming invariance in collectivistic contexts. While the multi-group CFA supported full measurement invariance across examined subgroups based on hierarchical model comparisons, we acknowledge important methodological considerations. Invariance conclusions are based primarily on changes in fit indices (ΔCFI ≤ 0.01, ΔRMSEA ≤ 0.015) between nested models rather than absolute model fit. Several subgroup models, particularly for dietary habits and physical activity levels, demonstrated RMSEA values exceeding conventional thresholds (ranging from 0.10 to 0.15 across subgroups), despite meeting invariance criteria through relative fit comparisons. This pattern reflects the inherent challenges of fitting brief instruments to multiple subgroups with smaller sample sizes. The invariance conclusions indicate that the factor structure and loadings function equivalently across groups, a prerequisite for valid group comparisons, but do not imply absolute model fit excellence within each subgroup. This distinction is important for interpretation: observed differences in latent means across groups reflect true differences in the construct rather than measurement artifacts, yet absolute fit statistics suggest some residual misspecification remains. Future research with larger subgroup samples may further refine understanding of subgroup-specific model fit.

The Arabic LSNS-6 demonstrated moderate positive correlations with the MOS-SSS subscales, supporting its convergent validity. The strongest associations were observed with overall perceived social support (r = 0.51) and Emotional/Informational Support (r = 0.48). The Family subscale showed consistently stronger correlations with all MOS-SSS dimensions (r = 0.45 to 0.52) compared to the Friends subscale (r = 0.29 to 0.41), particularly for Tangible and Affectionate Support. In contrast, both subscales were similarly correlated with Positive Social Interaction, which suggests that while family remains the primary source of instrumental and emotional support, friendships also contribute meaningfully to social engagement.

While the Arabic LSNS-6 and the MOS-SSS both assess aspects of social relationships and support, they capture complementary but distinct constructs, which helps explain the moderate correlations observed between them (r = 0.43–0.51 with MOS-SSS subscales; r = 0.51 with overall perceived social support). The LSNS-6 is a structurally oriented measure that quantifies the size and frequency of social network contacts (e.g., objective social connectedness with family and friends), whereas the MOS-SSS is functionally oriented, assessing the perceived availability and quality of different types of support (emotional/informational, tangible, affectionate, and positive social interaction). These conceptual differences, structural versus functional, naturally result in correlations that are positive and meaningful but not high enough to suggest redundancy.

The IRT analysis further elucidated the psychometric properties of the Arabic LSNS-6. Items 2 and 3 within the Family subscale had high discrimination values (>3.0), indicating that these items effectively differentiate individuals with varying levels of family support. Conversely, item 1 had notably lower discrimination (0.38), reinforcing the notion that frequency of contact may not be the most reliable indicator of meaningful social connections in this cultural context.

Within the Friends subscale, the discrimination values increased progressively across items 4, 5, and 6, with item 6 demonstrating the highest discrimination power (12.61). This suggests that the ability to rely on friends for practical help is a particularly strong indicator of overall social network quality. The difficulty parameters (b1–b5) for most items followed a logical progression, effectively covering a broad range of social network levels. However, reduced precision was observed at the upper end of the scale, indicating limited sensitivity in detecting individuals with very strong social networks. This could be due to a lack of challenging (or “difficult”) items that specifically assess richer or more complex social ties, making it harder to differentiate among individuals who are already highly socially connected. This suggests a potential need for refinement of the LSNS-6, possibly through the addition of more nuanced items, to improve measurement sensitivity at higher levels of social support. Finally, the covariance values suggest a moderate to strong relationship between the family and friend factors. While interconnected, these domains remain conceptually distinct and should be assessed separately to capture the multifaceted nature of social support.

The LCA revealed four meaningful and clinically interpretable profiles of social connectedness and perceived support among older Arabic-speaking adults, extending the findings from classical and IRT analyses. Approximately 60% of the sample belonged to well-supported classes (Class 1 and Class 3), characterized by strong overall networks and high affectionate/positive interaction support, which aligns with the cultural emphasis on familial collectivism previously observed in the sample. These groups would benefit primarily from maintenance-oriented strategies that preserve existing social ties and encourage continued participation in community and family activities. In contrast, the identification of a substantial vulnerable subgroup (Class 2, ≈18%) with particularly low emotional and affectionate support highlights an important at-risk population that may be overlooked when relying solely on total scores or mean comparisons. The emergence of a tangible-focused intermediate class (Class 4) further suggests heterogeneity in how older adults experience practical versus emotional support, potentially reflecting generational or situational differences in family roles. These findings reinforce the multidimensional nature of SIL in collectivistic contexts and provide a foundation for developing targeted, profile-specific interventions to reduce SIL and its associated health risks.

### 4.1. Implications for Research and Public Health Interventions

The validation of the Arabic LSNS-6 has important implications for both academic research and public health. From a research perspective, this study provides a culturally adapted and psychometrically validated tool for assessing social networks among Arabic-speaking older adults. Future research can use this instrument to explore the impact of SIL on various health outcomes. 

From a public health standpoint, the Arabic LSNS-6 can serve as a screening tool for identifying individuals at risk of SIL. Additionally, the observed cultural emphasis on family-centered support highlights both a strength and a potential vulnerability. Interventions should aim not only to strengthen existing family ties but also to encourage the development of broader social networks, especially among older adults.

The identification of meaningful social support profiles through LCA enhances the Arabic LSNS-6’s utility as a screening tool. Beyond identifying SIL via total scores, the scale can now inform stratified intervention approaches. Individuals in the low-support class (Class 2) would benefit from interventions emphasizing emotional connection and affective support, such as peer support groups, counseling services, or community programs fostering meaningful relationships. Those in the tangible-focused class (Class 4) might benefit from interventions strengthening emotional reciprocity within existing practical relationships. This profile-based approach represents an advance over aggregate screening, enabling health systems to allocate limited intervention resources toward populations with the greatest need for specific dimensions of social support.

### 4.2. Limitations

Despite its strengths, this study has several limitations that should be acknowledged. First, the reliance on self-reported measures introduces the possibility of response bias, as participants may overestimate or underestimate their social engagement. Future research should incorporate objective measures of social interaction (e.g., digital tracking of communication patterns, ecological momentary assessments) to improve measurement accuracy. 

Second, the sample was recruited entirely through online and social media platforms. This method may introduce selection bias, as participation depends on digital access, technological literacy, and engagement with online networks. Older adults who are less digitally connected may therefore be underrepresented. These factors should be considered when interpreting the findings, and future studies are encouraged to use multiple recruitment methods to reach individuals with limited access to digital technologies.

Third, the demographic characteristics of the sample may also limit the generalizability of the findings. The study predominantly included female participants (75%), which may have influenced the reported levels of social connectedness and support. Additionally, 66% of participants were unemployed or retired, which could impact the structure and availability of social networks. Older adults who are no longer in the workforce may experience reduced daily social interactions compared to those who are still employed. Future research should explore how employment status influences social network size and quality. 

Fourth, health-related factors also emerged as potential contributors to SIL. A significant proportion of participants reported poor or very poor mental health (31%), which aligns with existing literature on the link between SIL and psychological distress (Menec et al., 2020). Additionally, 56% of participants reported no regular physical activity, and 31% were taking regular medications; this suggests potential health-related barriers to maintaining an active social life. Future interventions should consider these health vulnerabilities when designing programs to enhance social connectedness. 

Fifth, although the Arabic LSNS-6 showed good internal consistency, this study did not assess test–retest reliability. Future research should evaluate the temporal stability of the instrument to ensure consistent measurement of social network characteristics over time. Measurement invariance across countries was also not assessed because the number of participants within each country subgroup was too small to support robust multi-group analyses. Future studies should test invariance in larger, more balanced country samples and explore additional factors such as socioeconomic status to improve generalizability and inform targeted public health strategies.

Finally, the weak factor loading of Item 1 (assessing contact frequency with relatives) suggests that this item may not optimally capture family support in the Arabic cultural context. Future refinement of this item, potentially through cognitive interviews or qualitative research, may enhance the scale’s cultural validity and measurement precision. 

## 5. Conclusions

In conclusion, the Arabic version of the LSNS-6 demonstrates strong reliability and validity for assessing SIL among older Arabic-speaking adults. The scale supports a robust two-factor structure distinguishing family and friendship networks and shows measurement invariance across key sociodemographic and lifestyle groups. The LCA further identified a vulnerable subgroup characterized by low social support, highlighting the scale’s utility beyond total scores. Although the instrument performs well overall, refinement of specific items may enhance its sensitivity, particularly at higher levels of social connectedness. Future research should examine test–retest reliability and responsiveness to change. From a public health perspective, the Arabic LSNS-6 represents a practical and culturally appropriate screening tool to support surveillance and guide targeted interventions aimed at strengthening both familial and non-familial social ties in aging populations.

## Figures and Tables

**Table 1:** Descriptive statistics of the sociodemographic variables of the study participants (N = 327).

Category	Counts	% of Total	95% CI
**Sex**			
Female	245	75.00%	70.4–79.6%
Male	82	25.00%	20.4–29.6%
**Marital Status**			
Not married	124	38.00%	33.0–43.0%
Married	203	62.00%	57.0–67.0%
**Living Arrangements**			
Alone	70	21.00%	16.4–25.6%
With others	257	79.00%	74.4–83.6%
**Job**			
Employed	111	34.00%	29.1–39.9%
Unemployed/Retired	216	66.00%	60.1–70.9%
**General Health**			
Average	93	28.00%	23.0–33.0%
Excellent	27	8.00%	5.3–12.0%
Good	142	43.00%	37.6–48.4%
Poor	5	2.00%	0.6–4.3%
Very Good	60	18.00%	14.0–22.9%
**Cognitive Health**			
Average	101	31.00%	26.1–36.0%
Excellent	26	8.00%	5.1–12.3%
Good	127	39.00%	34.3–43.9%
Poor	61	19.00%	15.3–23.6%
Very poor	12	4.00%	2.1–7.2%
**Mental Health**			
Average	143	44.00%	38.9–49.1%
Excellent	13	4.00%	1.9–8.5%
Good	69	21.00%	16.5–25.9%
Poor	72	22.00%	17.6–26.7%
Very Poor	30	9.00%	6.0–13.2%
**Physical Health**			
Average	115	35.00%	30.1–40.2%
Excellent	19	6.00%	3.3–10.1%
Good	107	33.00%	28.0–38.2%
Poor	74	23.00%	18.3–27.9%
Very Poor	12	4.00%	2.1–7.2%
**Diet Consistency**			
Sometimes	121	37.00%	32.1–42.0%
Unwatchful	105	32.00%	27.0–37.4%
Watchful	101	31.00%	25.8–36.3%
**Engagement in Physical Activity**			
Daily	33	10.00%	6.8–14.8%
None	183	56.00%	50.1–61.8%
On weekly basis	111	34.00%	29.1–39.9%
**Regular Medications**			
No	227	69.00%	64.2–73.5%
Yes	100	31.00%	26.5–36.1%

**Notes:** Results are expressed as absolute counts, corresponding percentages and 95% confidence intervals. The mean age of participants was 63.05 years, median of 63, SD of 4.42, IQR of 8.00.

**Table 2:** Descriptive statistics of the study variables of the study participants (N = 327).

Variable	Mean	Median	SD	IQR	Skewness	Kurtosis
LSNS-6 item 1	3.66	4	1.1	1	−0.92	0.32
LSNS-6 item 2	2.09	2	1.32	2	0.04	−0.73
LSNS-6 item 3	2.31	2	1.27	1	−0.29	−0.74
LSNS-6 item 4	2.36	3	1.34	2	−0.1	−0.66
LSNS-6 item 5	1.8	2	1.23	2	0.14	−0.83
LSNS-6 item 6	1.83	2	1.16	2	0.07	−0.7
LSNS-6 Family	8.06	8	2.87	4	−0.15	−0.51
LSNS-6 Friends	5.98	6	3.31	6	−0.07	−0.46
**LSNS-6 Total**	14.04	14	5.46	8	0.01	−0.27
Emotional/informational support	28.84	31	9.71	14.5	−0.61	−0.79
Tangible support	14.15	15	4.73	8	−0.39	−0.86
Affectionate support	10.24	11	3.85	7	−0.36	−1.13
Positive social interaction	9.89	10	3.57	7	−0.15	−1.08
**Overall social support**	66.38	66	20.52	31	−0.39	−0.77

**Notes:** LSNS-6: Lubben Social Network Scale (6 items). MOS: Medical Outcomes Study. LSNS-6 Family: Sum of LSNS-6 item 1, LSNS-6 item 2, and LSNS-6 item 3. LSNS-6 Friends: Sum of LSNS-6 item 4, LSNS-6 item 5, and LSNS-6 item 6. LSNS-6 Total: Sum of LSNS-6 Family and LSNS-6 Friends. Emotional/informational support: Sum of MOS-SSS items 2, 3, 7, 8, 12, 15, 16, and 18. Tangible support: Sum of MOS-SSS items 1, 4, 11, and 14. Affectionate support: Sum of MOS-SSS items 5, 9, and 19. Positive social interaction: Sum of MOS-SSS items 6, 10, and 17. Overall social support: Sum of all 19 MOS items.

**Table 3:** Internal consistency reliability of the Arabic LSNS-6 and its subscales (N = 327).

Statistic	McDonald’s ω	Cronbach’s α	Guttman’s λ2	Greatest Lower Bound
**LSNS-6 Family**				
Point estimate	0.75	0.70	0.72	0.75
95% CI lower bound	0.71	0.60	0.67	0.71
95% CI upper bound	0.80	0.75	0.76	0.79
If item dropped	McDonald’s ω	Cronbach’s α	Guttman’s λ2	Greatest Lower Bound
LSNS-6 item 1	N/A	0.83	0.83	0.83
LSNS-6 item 2	N/A	0.40	0.40	0.40
LSNS-6 item 3	N/A	0.32	0.32	0.32
**LSNS-6 Friends**				
Point estimate	0.86	0.86	0.86	0.86
95% CI lower bound	0.83	0.83	0.82	0.82
95% CI upper bound	0.89	0.88	0.89	0.89
If item dropped	McDonald’s ω	Cronbach’s α	Guttman’s λ2	Greatest Lower Bound
LSNS-6 item 4	N/A	0.75	0.75	0.75
LSNS-6 item 5	N/A	0.87	0.87	0.87
LSNS-6 item 6	N/A	0.79	0.79	0.79
**LSNS-6 Total**				
Point estimate	0.84	0.83	0.84	0.90
95% CI lower bound	0.81	0.80	0.81	0.89
95% CI upper bound	0.86	0.86	0.87	0.92
If item dropped	McDonald’s ω	Cronbach’s α	Guttman’s λ2	Greatest Lower Bound
LSNS-6 item 1	0.86	0.86	0.87	0.92
LSNS-6 item 2	0.81	0.79	0.81	0.85
LSNS-6 item 3	0.81	0.78	0.81	0.85
LSNS-6 item 4	0.80	0.79	0.81	0.88
LSNS-6 item 5	0.80	0.78	0.80	0.88
LSNS-6 item 6	0.79	0.78	0.79	0.86

**Notes:** LSNS-6: Lubben Social Network Scale (6 items). MOS: Medical Outcomes Study. LSNS-6 Family: Sum of LSNS-6 item 1, LSNS-6 item 2, and LSNS-6 item 3. LSNS-6 Friends: Sum of LSNS-6 item 4, LSNS-6 item 5, and LSNS-6 item 6. LSNS-6 Total: Sum of LSNS-6 Family and LSNS-6 Friends. N/A: Not applicable to compute.

**Table 4:** Confirmatory factor analysis of the LSNS-6 (N = 327).

Factor	Factor Loading (Standardized)					Residual Variance			
	Item	Estimate	SD	z-Value	*p*	Estimate	SD	z-Value	*p*
LSNS-6 Family	LSNS-1	0.26	0.07	4.47	<0.001	0.93	0.09	12.61	<0.001
	LSNS-2	0.84	0.07	16.41	<0.001	0.29	0.09	5.83	<0.001
	LSNS-3	0.85	0.07	16.45	<0.001	0.28	0.08	5.73	<0.001
LSNS-6 Friends	LSNS-4	0.72	0.07	14.47	<0.001	0.48	0.08	11.10	<0.001
	LSNS-5	0.84	0.06	17.86	<0.001	0.30	0.05	8.62	<0.001
	LSNS-6	0.92	0.05	20.27	<0.001	0.16	0.04	4.93	<0.001

Notes: LSNS-6 = Lubben Social Network Scale (6 items); MOS = Medical Outcomes Study. LSNS-6 Family = items 1–3; LSNS-6 Friends = items 4–6. CFA showed a significantly better fit for the two-factor model (χ^2^ = 41.53, df = 8, *p* < 0.001) compared with the baseline model (χ^2^ = 928.462, df = 15). Fit indices indicated good model fit (CFI = 0.963; TLI/NNFI = 0.931; NFI = 0.955; PNFI = 0.509; RFI = 0.916; IFI = 0.964; SRMR = 0.03; RMSEA = 0.113, 90% CI [0.081–0.148], *p* = 0.001; GFI = 0.992; MFI = 0.95). Information criteria were AIC = 5542.582, BIC = 5614.591, SSABIC = 5554.324 (log-likelihood = −2752.291; 19 parameters). Sampling adequacy was acceptable (KMO = 0.754; item KMO = 0.709–0.803; Bartlett’s χ^2^ = 917.577, df = 15, *p* < 0.001). Convergent and discriminant validity were supported (AVE: Family = 0.544, Friends = 0.673; HTMT = 0.712).

**Table 5:** Convergent validity of the LSNS-6 with the Medical Outcomes Social Support (MOS-SS) (N = 327).

Variable	LSNS-6 Family	LSNS-6 Friends	LSNS-6 Total	Emo./Info. Support	Tang. Support	Aff. Support	Pos. Social Interaction	Overall MOS-SSS
**LSNS-6 Family**	-							
**LSNS-6 Friends**	r = 0.56 *p* < 0.001	-						
**LSNS-6 Total**	r = 0.87 *p* < 0.001	r = 0.90 *p* < 0.001	-					
**Emo./Info. Support**	r = 0.46 *p* < 0.001	r = 0.38 *p* < 0.001	r = 0.48 *p* < 0.001	-				
**Tang. Support**	r = 0.51 *p* < 0.001	r = 0.29 *p* < 0.001	r = 0.45 *p* < 0.001	r = 0.65 *p* < 0.001	-			
**Aff. Support**	r = 0.49 *p* < 0.001	r = 0.29 *p* < 0.001	r = 0.43 *p* < 0.001	r = 0.69 *p* < 0.001	r = 0.71 *p* < 0.001	-		
**Pos. Social Interaction**	r = 0.45 *p* < 0.001	r = 0.41 *p* < 0.001	r = 0.48 *p* < 0.001	r = 0.74 *p* < 0.001	r = 0.72 *p* < 0.001	r = 0.84 *p* < 0.001	-	
**Overall MOS-SSS**	r = 0.52 *p* < 0.001	r = 0.39 *p* < 0.001	r = 0.51 *p* < 0.001	r = 0.93 *p* < 0.001	r = 0.83 *p* < 0.001	r = 0.87 *p* < 0.001	r = 0.90 *p* < 0.001	-

**Notes:** LSNS-6: Lubben Social Network Scale (6 items). MOS: Medical Outcomes Study. LSNS-6 Fam. = LSNS-6 Family. LSNS-6 Fr. = LSNS-6 Friends. LSNS-6 Tot. = LSNS-6 Total. Emo./Info. Sup. = Emotional/Informational Support. Tang. Sup. = Tangible Support. Aff. Sup. = Affectionate Support. Pos. Soc. Int. = Positive Social Interaction. Overall MOS-SSS = Overall Social Support as per Medical Outcomes Study.

**Table 6:** Multi-item response theory of the LSNS-6 (N = 327).

Item	a1	a2	b1	b2	b3	b4	b5	cov.F1	cov.F2
LSNS-1	0.38	0	−6.88	−4.65	−1.86	3.77	2.30	1	0.67
LSNS-2	3.45	0	−1.17	−0.55	0.26	1.18	2.28	0.67	1
LSNS-3	3.64	0	−1.29	−0.78	0.07	0.9	2.96	1	0.67
LSNS-4	0	2.06	−1.57	−0.66	0.1	1.27	2.21	0.67	1
LSNS-5	0	3.33	−1.01	−0.19	0.49	1.58	2.92	1	0.67
LSNS-6	0	12.61	−0.99	−0.17	0.44	1.59	2.65	0.67	1

**Notes:** LSNS-6: Lubben Social Network Scale (6 items). MOS: Medical Outcomes Study. LSNS-6 Family: Sum of LSNS-6 item 1, LSNS-6 item 2, and LSNS-6 item 3. LSNS-6 Friends: Sum of LSNS-6 item 4, LSNS-6 item 5, and LSNS-6 item 6. The a1 and a2 represent loadings for Factor 1 and Factor 2, respectively. b1 to b5 represent the difficulty parameters for the items. The Means column indicates the average scores for each item. cov.F1 and cov.F2 represent the covariances with Factor 1 and Factor 2. A value of 1.00 indicates a perfect correlation, while values below 1.00 indicate varying degrees of correlation.

**Table 7:** Interpretation of the four-class latent class solution for social connectedness and perceived social support (N = 327).

Class	Label/Interpretation	Key Characteristics (Dominant Response Patterns)	Typical LSNS-6 Total Range (Approximate)
1	Well-connected/High-support	Highest probabilities across LSNS-6 total and all MOS-SSS domains (strong emotional, tangible, affectionate, and positive interaction support)	Mid-high (≈14–22+)
2	Relatively isolated/Weak emotional support	Lowest probabilities on emotional/informational and affectionate support; generally lower LSNS-6 scores	Low–mid (≈8–14)
3	Family-centered/High affectionate support and interaction	Very high affectionate support and positive social interaction; moderate–high tangible support	Mid (≈12–18)
4	Pragmatic/Tangible-focused intermediate	Relatively strong tangible support; moderate emotional/informational; lower positive social interaction	Mid (≈12–18)

## Data Availability

Data associated with this paper can be produced on request from the corresponding author.

## Supplementary Materials

The following supporting information can be downloaded at: https://www.mdpi.com/article/10.3390/ejihpe16030040/s1, Table S1. Model fit statistics by group.
