# Supplementary material for: The Arabic Lubben Social Network Scale-6: Psychometric Validation, Measurement Invariance, and Social Support Profiles in Arabic-Speaking Older Adults
Source: Eur J Investig Health Psychol Educ. 2026 Mar 6;16(3):40. doi: 10.3390/ejihpe16030040 (PMC13025865; doi:10.3390/ejihpe16030040)
Supplement: Supplementary file 1 [file ejihpe-16-00040-s001.zip › ejihpe-4100127-supplementary.pdf]

Table S1. Model Fit Statistics by Group

Group	χ^2	df	p-value	CFI	TLI	RMSEA	RMSEA 90% CI LL	RMSEA 90% CI UL
**Female**	33.51	8	< .0001	0.96	0.93	0.11	0.08	0.16
**Male**	17.47	8	0.0255	0.96	0.92	0.12	0.04	0.20
**Married**	41.53	8	< .0001	0.96	0.93	0.11	0.08	0.15
**Not Married**	41.53	8	< .0001	0.96	0.93	0.11	0.08	0.15
**Living with others**	41.53	8	< .0001	0.96	0.93	0.11	0.08	0.15
**Living alone**	41.53	8	< .0001	0.96	0.93	0.11	0.08	0.15
**Watchful diet**	28.05	8	0.0005	0.94	0.89	0.16	0.10	0.22
**Diet sometimes**	16.30	8	0.0383	0.97	0.94	0.09	0.02	0.16
**Physical activity: none**	22.68	8	0.0038	0.97	0.94	0.10	0.05	0.15
**Physical activity: weekly**	27.31	8	0.0006	0.95	0.90	0.15	0.09	0.21
**Physical activity: daily**	7.93	8	0.4401	1.00	1.00	0.00	0.00	0.20
